# MicroRNA Let-7 in B lymphocyte activation

**DOI:** 10.18632/aging.101968

**Published:** 2019-05-11

**Authors:** Shuai Jiang, Wei Yan, Shizhen Emily Wang

**Affiliations:** 1 Department of Pathology, University of California, San Diego, La Jolla, CA 92093, USA; 2 Division of Biology and Biological Engineering, California Institute of Technology, Pasadena, CA 91125, USA

**Keywords:** MicroRNA let-7 cluster, B cell activation, glucose and glutamine availability, Lin28a, antibody production

Over the past twenty years, mounting evidence showed that microRNA (miR) plays indispensable roles in various biological processes including aging process, immune cell responses and metabolic reprogramming through posttranscriptional gene targeting [1]. MiR-encoding genes are distributed in different chromosomes as individual genes or in clusters in human and murine genome [1]. Each miR cluster encodes at least two miRs that sometimes belong to the same family, making it critical to dissect the physiological and pathological roles of each clustered miR vs. the entire gene cluster in a given cellular context, such as B cells. Let-7 was one of the two ancient miRs initially found in *C. elegans* as a regulator of developmental timing [2]. The Let-7 family has twelve members which are distributed on seven different chromosomes in murine genome. Interestingly, all twelve members share the same “seed sequence”, which is key to the complementation between miR and its target genes [3]. An intriguing question is why evolutionarily there are so many members in the let-7 family sharing the exactly same seed sequence. Do they play individual or redundant roles in various cellular context? Our recent work using transgenic mouse models of different let-7 family members revealed that some let-7 miRs express widely in differentiated immune cells including activated splenic B cells (4). We found that let-7a, let-7d, and let-7f were induced by LPS in splenic B cells, and that the let-7adf cluster inhibited B cell activation, whereas let-7e and let-7g were significantly decreased by LPS [4]. Based on these findings, we speculate that let-7e and let-7g might have unique functions compared to the let-7adf cluster in activated splenic B cells. Future experiments with overexpression or deletion of singular let-7e or let-7g by using engineered mouse models are essential to determine their physiological roles in B cells.

**Figure 1:**
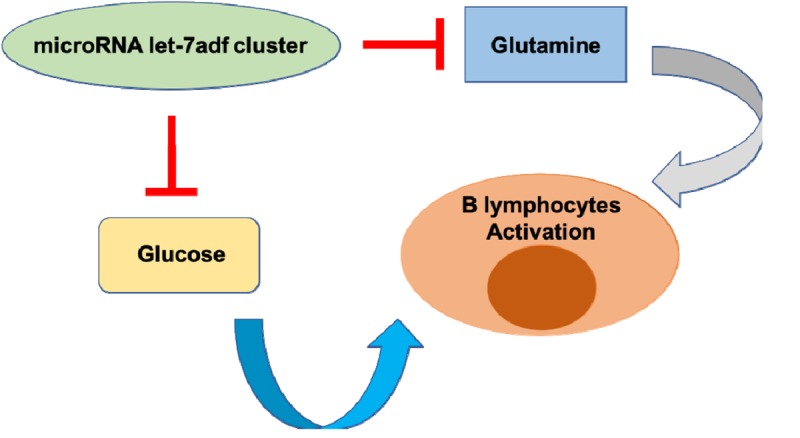
**MicroRNA** **let-7adf: A metabolic checkpoint of B lymphocyte activation**. A specific cluster of microRNA let-7 family suppresses B lymphocyte activation by restricting metabolic rates of glucose and glutamine.

Glucose and glutamine are two principle nutrients in B cell activation, proliferation, and differentiation. Regulation of glucose and glutamine metabolism plays a key role in B cell activation [5]. Our study for the first time reported that both uptake and utilization of glucose and glutamine could be controlled by the let-7adf cluster (Figure 1) [4]. However, it is not clear which let-7 member individually regulates metabolism of the two major nutrients. Future work using singular let-7a-1, let-7d and let-7f-1 KO/iTg engineered mouse models would help clarify the complex regulatory network in B cell activation.

We reported that comprehensive repression of all let-7 members by overexpressing Lin28a in B cells contributes to both T cell-independent (TI) and T cell-dependent (TD) antigen-induced antibody production [4]. Lin28a acts as both a RNA-binding protein and inhibitor of let-7 bioprocessing in cancer and immune cells [6]. Our study indicated that other let-7 members, except let-7b and let-7c2, might contribute to TD antigen-induced antibody production, and it is likely that Lin28a might modulate TD responses by directly binding to certain key enzymes in activated B cells [4]. This is consistent with the finding that Lin28a could directly bind to and enhance the translation of metabolic enzymes such as Pfkp in ear tissues and mouse embryonic fibroblasts [7].

Future studies using profiling technologies such as RNA immunoprecipitation sequencing and liquid chromatography-tandem mass spectrometry would provide a global picture of miR-mediated gene and metabolic regulations in anti-CD40-activated Lin28a iTg B cells.

Although we showed that the let-7adf cluster is essential to TI responses in splenic B cells by tracing and analyzing both glucose and glutamine metabolism [3], many questions remain unanswered. For example, do other major metabolic pathways including fatty acid oxidation (FAO) and pentose phosphate pathway contribute to let-7adf-mediated splenic B cell activation? Is the let-7adf cluster or other let-7 clusters important for the activation of other B cell subtypes? It could be important to analyze and compare the metabolome of different types of B cells. Metabolic checkpoint regulators in other B cell subtypes, such as GSK3 that functions as a metabolic sensor in germinal center (GC) B cells, have just started to be identified [8]. It would be of great interest to investigate the post-transcriptional regulations of GC B cell metabolism by miRs by using the KO/iTg engineered mouse models.

Our study focused on the physiological role of let-7adf cluster in B cell activation in young adult mice [3], but it would be worthwhile to determine the pathological roles of this cluster in B cell functions in aging mice or by challenging the let-7adf KO/iTg mice with autoimmune diseases models. These future studies may provide a comprehensive understanding of B cell biology and provide clinical implications for miR-let-7adf-targeted therapy.
